# Aesthetic Surgery Gone Wrong: A Case Report and Literature Review of Acute Kidney Injury Secondary to Hematoma After Liposuction

**DOI:** 10.7759/cureus.39820

**Published:** 2023-06-01

**Authors:** Kateryna Georgiyeva, Daniel Shlyak, Francisco Duarte, Harendra Kumar, John Sciarra

**Affiliations:** 1 Internal Medicine, Memorial Healthcare System, Pembroke Pines, USA; 2 Anesthesiology, Larkin Community Hospital, South Miami, USA; 3 Critical Care Medicine, Larkin Community Hospital, South Miami, USA; 4 Medicine and Surgery, Dow University of Health Sciences, Karachi, PAK; 5 Anesthesiology, Larkin Community Hospital, South Miami, USA

**Keywords:** perioperative complications, abdominal hematoma, plastic and reconstructive surgery, liposuction complications, : acute kidney injury

## Abstract

Liposuction is a widely used cosmetic surgery that involves the removal of excess fatty tissue. Although it is generally considered a safe and effective procedure, complications can arise. Acute kidney injury (AKI) is a serious complication, which can be caused by various factors. Extravasation of blood from vessels damaged by the cosmetic liposuction procedure cause hypovolemia and intravascular depletion, significant factors leading to pre-renal acute kidney injury. In this case report, we present the case of a 29-year-old female patient who developed AKI after undergoing a liposuction and "Brazilian Butt Lift (BBL)" procedure. The patient experienced persistent nausea, vomiting, and abdominal pain postoperatively and was admitted to the ICU. The patient's condition gradually worsened over the next few days, and imaging of the abdomen revealed a complex, clotted hematoma in abdominal and pelvic cavities that required surgical intervention. Her care involved a collaborative effort from critical care, plastic surgery, and nephrology specialists. This case highlights the potential complications of cosmetic surgery and the need for comprehensive postoperative care to manage these complications effectively. It also emphasizes the importance of identifying and managing risk factors for AKI during liposuction to minimize the risk of this serious complication.

## Introduction

Aesthetic surgery has increased in popularity worldwide, with millions of people seeking to enhance their physical appearance via various procedures. While many of the aesthetic procedures and cosmetic surgeries are safe and effective, they do come with associated risks and post-operative complications which can be severe and life-threatening such as acute kidney injury (AKI) [1,2].

AKI is a rare but severe consequence of liposuction and Brazilian Butt Lift (BBL) procedures, which are among the most often done cosmetic operations worldwide [3]. Fluid alterations, electrolyte imbalances, and renal ischemia are likely to play a role in the pathophysiology of AKI in these individuals. The incidence of AKI following these surgeries is expected to occur in less than 1% of patients, yet it poses a significant health deterioration risk [1,3]. 

The Acute Dialysis Quality Initiative (ADQI) is a global partnership of specialists working in the field of AKI. ADQI attempts to provide consensus guidelines and recommendations to improve AKI care and results. The prevention, diagnosis, and treatment of perioperative AKI is one area of focus for ADQI [1,3,4].

Perioperative AKI is defined as an acute kidney injury that happens during the perioperative period, which includes the time before, during, and after surgery. It is a significant consequence that may result in increased morbidity, mortality, and healthcare costs. ADQI has developed consensus guidelines on the diagnosis and management of perioperative AKI, which may aid healthcare practitioners in providing the best possible care to their patients [3,4].

There are three types of risk factors for perioperative AKI: preoperative, intraoperative, and postoperative variables. Advanced age, previous chronic kidney disease (CKD), diabetes mellitus, hypertension, congestive heart failure, liver disease, and certain medications (such as nonsteroidal anti-inflammatory drugs [NSAIDs] and angiotensin-converting enzyme inhibitors) are all risk factors for surgery [1,4,5]. These conditions may impair renal function and make patients more susceptible to AKI during the perioperative period [5].

In this case report, the patient was a 29-year-old female patient who developed AKI after liposuction and BBL procedures. The patient presented to the emergency department complaining of nausea, vomiting, stomach pain, and poor oral intake, all attributed to the procedure. Despite early intravenous fluids and ketorolac (an NSAID) administration for pain, the patient's condition deteriorated, and she eventually required surgical intervention to treat a complex, occluded hematoma.

This case demonstrates the risk of substantial difficulties after aesthetic surgery and the need for careful patient selection, preoperative evaluation, and postoperative therapy. It also emphasizes the need to raise awareness among healthcare practitioners and patients about the potential risks associated with these procedures. Given cosmetic surgery's growing popularity, healthcare practitioners must be aware of potential issues and collaborate closely with patients to ensure safe and successful outcomes.

## Case presentation

Ms. S, a 29-year-old female patient, presented to the emergency department (ED) complaining of persistent nausea, vomiting, abdominal pain, and poor oral intake. The patient had no significant medical history and had never been admitted to our hospital before. Upon arrival, her vital signs were stable, with a blood pressure of 108/55 mmHg, a pulse rate of 83 beats per minute, a respiratory rate of 18 breaths per minute, a temperature of 99°F, and an oxygen saturation of 100% on room air. In the ED, the patient was given ketorolac 30 mg and ondansetron before being cleared by the ED team and admitted directly to the ICU. Further examination revealed that the patient had undergone liposuction and BBL procedures three days before presentation, after which she experienced a loss of appetite, nausea, vomiting, and abdominal pain.

The KDIGO (Kidney Disease: Improving Global Outcomes) criteria for acute kidney injury was used to diagnose the patient with AKI upon the first day of admission to the ICU. According to KDIGO criteria, the patient’s AKI upon admission was stage 3, with her urine output being below <0.5 ml/kg/hr for the first 24 hours of admission to the hospital. Due to acute kidney injury, the patient was started on a clear liquid diet and intravenous fluids with lactated Ringer's (LR) solution at 100 ml/hour, and the nephrology team was consulted. Laboratory tests revealed elevated blood urea nitrogen (BUN) and creatinine levels, indicating worsening AKI, as well as low hemoglobin levels, for which she received packed red blood cell transfusions. Nephrology recommended intravenous fluid maintenance with LR and bumetanide 2 mg once daily to maintain urine output above 0.5 ml/kg/hr to avoid worsening of AKI in the oliguric phase, which was monitored with a Foley catheter. The patient’s urinalysis results on the first day of the hospital stay (admission day) and on the 11th day of hospital stay (a day before discharge) are shown in Table 1 below. The blood urea nitrogen/creatinine ratio, glomerular filtration rate, and 24-hour urine output levels on the first, sixth, and 12th days of the patient's hospital stay are shown in Table 2 below.

**Table 1:** Urinalysis results on the first day of the hospital stay (admission day) and on the 11th day of hospital stay (a day before discharge).

Urinalysis	Result Day 1 (Admission)	Result Day 11 (1 Day before Discharge)	Reference Range
Color	Yellow	Yellow	Yellow
Appearance	Clear	Clear	Clear
Specific Gravity	<1.005	1.2	1.005–1.030
Urine pH	6	6.5	4.5–8.0
Nitrite	Negative	Negative	Negative
Glucose	Negative	Negative	Negative
Ketone	Negative	Negative	Negative
Protein	2+	Negative	Negative
Blood	3+	Negative	Negative
Bilirubin	Negative	Negative	Negative
Urobilinogen	0.6	0.4	<2.0 mg/dL
Leukocyte Esterase	Negative	Negative	Negative
Urinalysis Microscopy	RBC casts, Hyaline casts	None seen	None seen
Red Blood Cells	5-10	0-2	0-2/hpf
White Blood Cells	3-5	0-2	0-5/hpf
Squamous Epithelium	Moderate	None seen	None seen
Bacteria	None seen	None seen	None seen

**Table 2:** BUN/creatinine ratio, GFR, and 24-hour urine output levels of the patient throughout various days of the hospital stay. BUN - Blood Urea Nitrogen GFR - Glomerular Filtration Rate

BUN/Creatinine Ratio		
	Result	Reference Range
Day 1 (Admission)	7	7 - 25
Day 6	9	7 - 25
Day 12 (Discharge)	17	7 - 25
GFR		
	Result	Reference Range
Day 1 (Admission)	45	≥60 mL/min/1.73 m²
Day 6	55	≥60 mL/min/1.73 m²
Day 12 (Discharge)	80	≥60 mL/min/1.73 m²
24-hour Urine Output		
	Result	Reference Range
Day 1 (Admission)	.2 ml/kg/hr	>0.5 ml/kg/hr
Day 6	1.6 ml/kg/hr	>0.5 ml/kg/hr
Day 12 (Discharge)	1.2 ml/kg/hr	>0.5 ml/kg/hr

**Figure 1:**
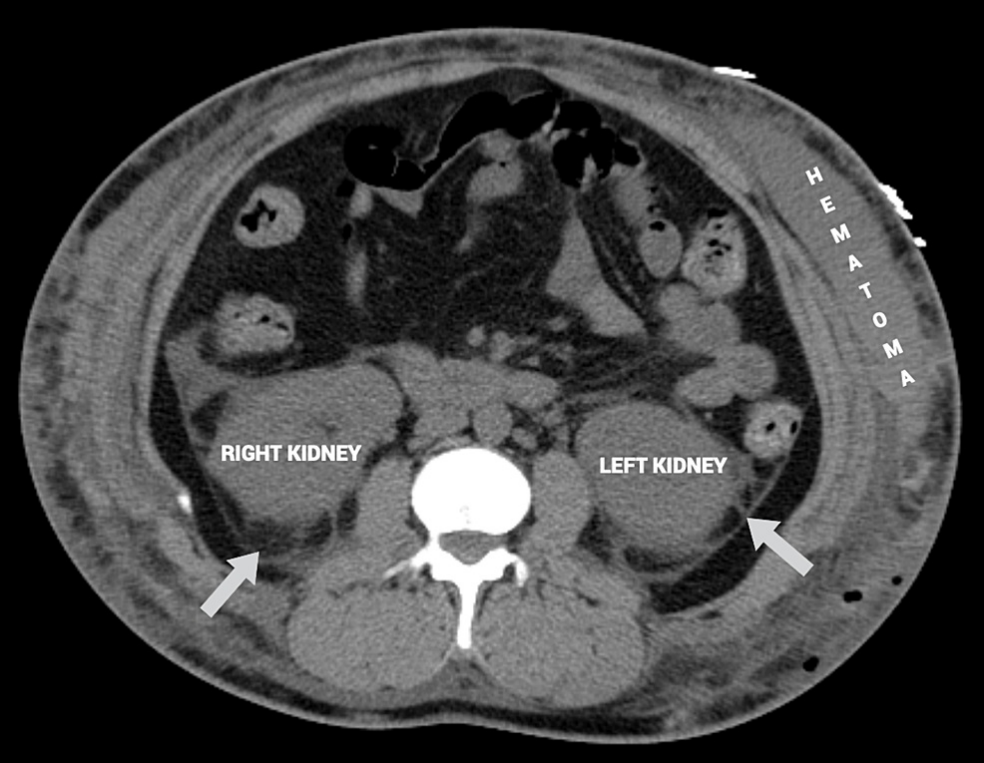
Computed tomography axial view. Perinephric fat stranding around both kidneys is shown (arrows). Hyperdense hematoma fluid collection on left side is shown. Perinephric - adjacent to the kidney

**Figure 2:**
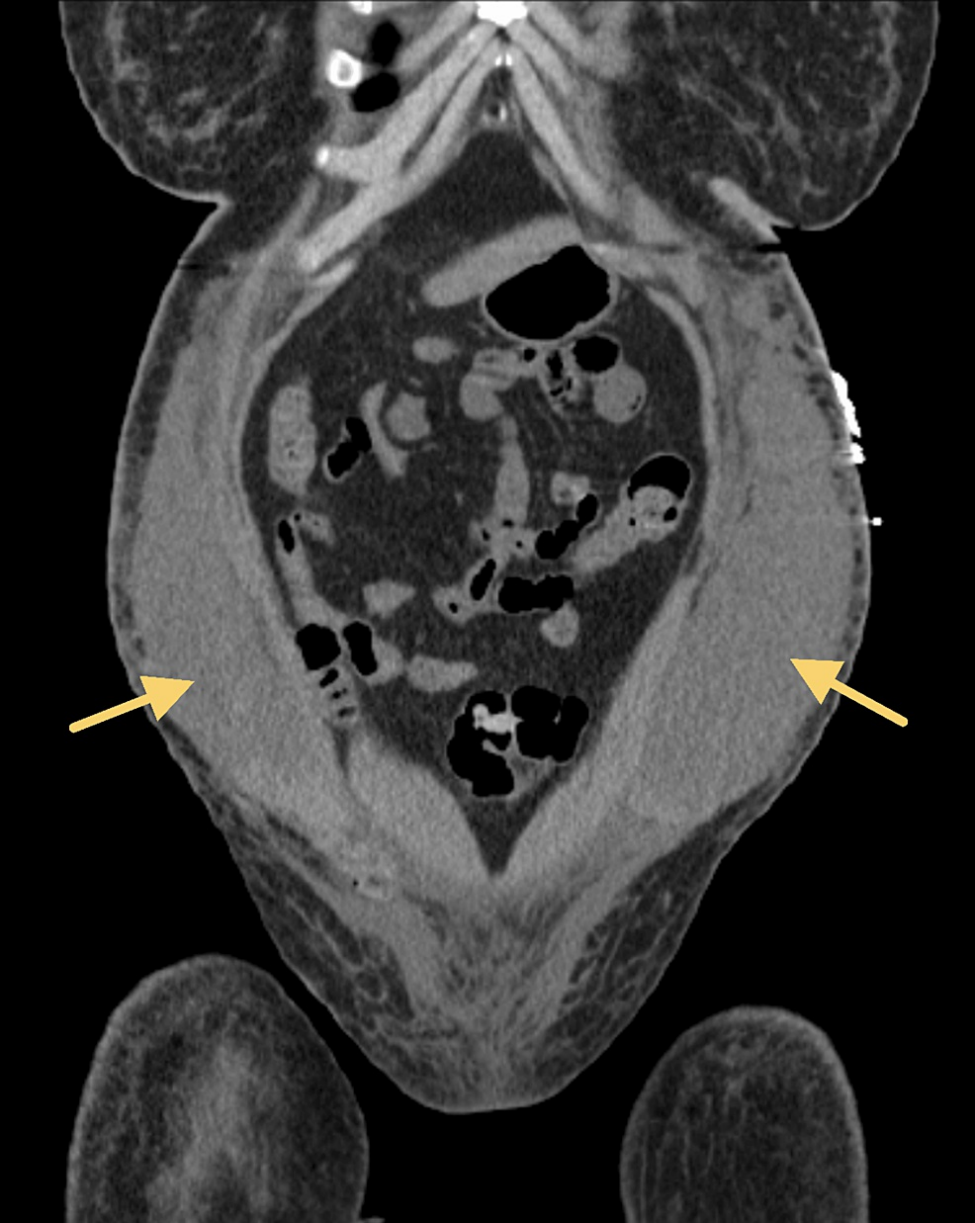
Computed tomography coronal view of hematoma, intra-abdominal and pelvic free fluid in abdominal cavity bilaterally (arrows).

**Figure 3:**
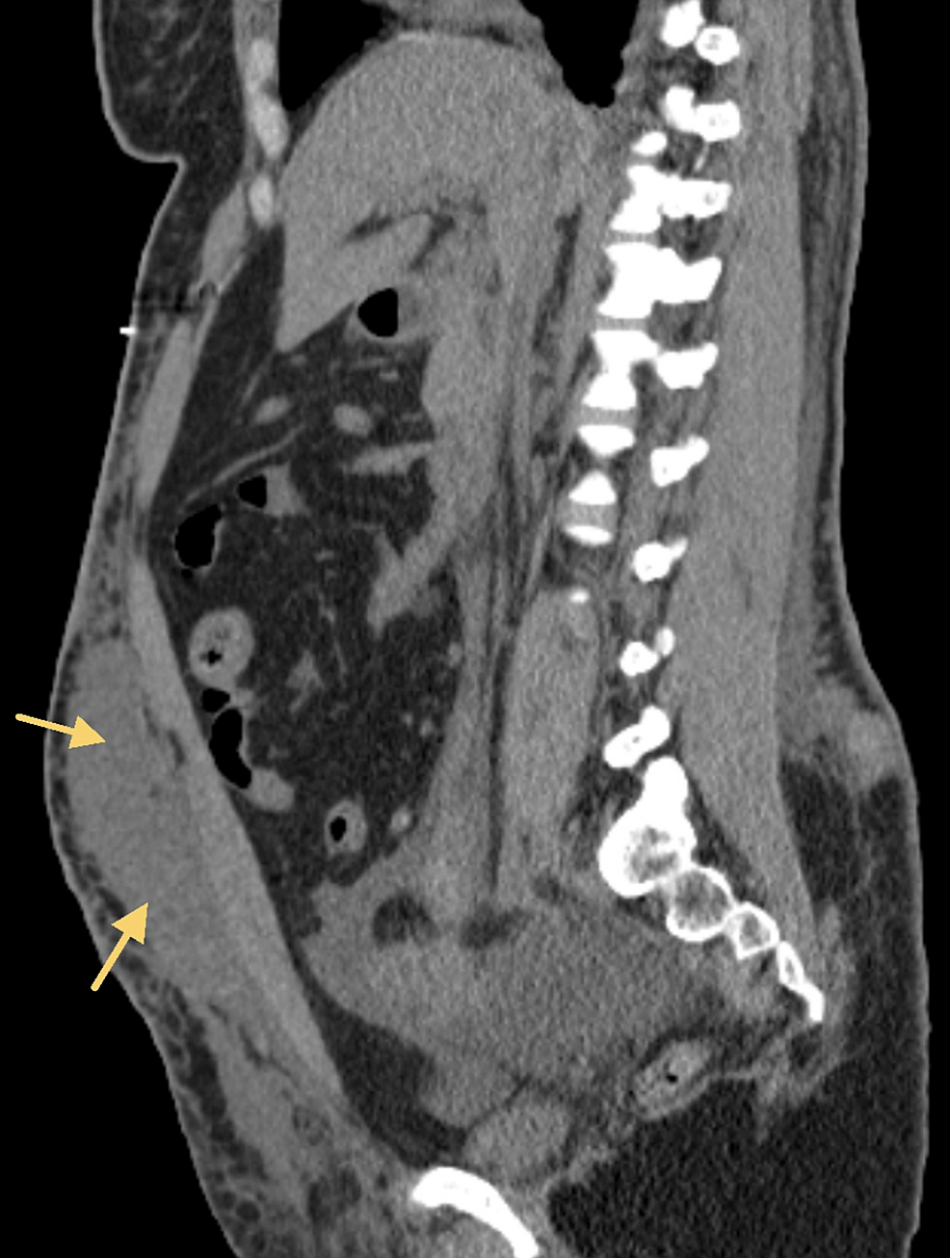
Computed tomography sagittal view of hematoma in abdominal and pelvic cavities bilaterally (arrows).

Despite being managed with pain medications such as morphine and hydromorphone, the patient continued to experience moderate to severe abdominal pain, persistent nausea and vomiting, and poor appetite. The abdominal pain in particular had worsened by the fifth day of admission. Imaging of the abdomen revealed a complex, clotted hematoma, as well as intra-abdominal and pelvic free fluid. Despite the risks, the plastic surgery, critical care, and anesthesiology teams agreed that surgical intervention was necessary. Plastic surgery performed an abdominal wall hematoma incision and debridement, as well as wound vac placement. The patient received two units of packed red blood cells intraoperatively, and her hemoglobin/hematocrit levels were checked again after surgery. Figure 1 demonstrates a computed tomography axial view scan of the patient's lower abdomen, and shows perinephric fat stranding [1] as well as the hematogenous free fluid collection. Figures 2, 3 demonstrate computed tomography coronal and sagittal views of the hematoma found in the patient's abdominal and pelvic cavities, respectively.

Despite adequate pain management, the patient's hospital stay was complicated by stage 3 AKI and persistent abdominal pain. The patient's condition gradually improved with continued medical and surgical management, and she was eventually discharged from the hospital after a 12-day stay. 

## Discussion

This case report demonstrates the potential hazards associated with cosmetic surgery, particularly liposuction. The patient in question had an acute kidney injury, anemia, and a complex hematoma that required surgical intervention. These issues necessitated a collaborative effort from various medical teams, resulting in a difficult recovery and a prolonged ICU stay.

Acute kidney injury is a potentially fatal condition that occurs when the kidneys suddenly stop functioning properly [2]. This may be caused by a variety of factors, including decreased blood flow to the kidneys, renal damage, or urinary system obstruction. In the case of the this patient, AKI was most likely caused by the formation of a hematoma, or collection of blood, in the abdominal wall during liposuction [2,3].

One of the most critical issues was AKI. AKI occurs when the kidneys abruptly lose their ability to filter waste from the blood. In this case, AKI was most likely caused by hypovolemia and intravascular depletion, which resulted in decreased renal perfusion. Liposuction involves the removal of large amounts of adipose tissue, which may cause significant fluid shifts and result in hypovolemia. The patient's renal perfusion may have been compromised as a consequence of the prolonged hypovolemia, culminating in AKI. Furthermore, the use of anesthesia and medications during surgery may result in AKI.

Moreover, anemia necessitated repeated blood transfusions. The cause of anemia in this case was most likely multifaceted. Anemia might have been caused by blood loss during the procedure and subsequent postoperative bleeding into the hematoma. The patient may also have seen a decrease in erythropoietin production, which is a hormone produced by the kidneys that promotes the formation of red blood cells. Given the patient's AKI, it is likely that erythropoietin production was restricted, resulting in anemia.

Another remarkable finding in this case was the complex hematoma seen on imaging. Hematomas are a common liposuction side effect that may cause significant morbidity and mortality, especially if infected. Hematomas form when blood collects outside of a blood vessel, forming a clot. The hematoma in this case was most likely caused by the surgery's prolonged hypovolemia and blood loss. The hematoma demanded surgical intervention, which included incising and debriding the affected area. This kind of surgery is considered the gold standard for dealing with complex hematomas.

Hematoma formation is a common side effect after liposuction, particularly in the abdominal region. The use of tumescent fluid, which is injected into the suctioned area, may result in bleeding and the formation of a hematoma [4]. Hematomas may cause compression of nearby tissues, such as blood vessels and organs, resulting in decreased blood flow and oxygen availability [4,5]. In this case, the hematoma most likely caused renal artery compression, resulting in decreased blood flow and, eventually, AKI.

Dehydration, hypotension, and the use of certain medications, such as NSAIDs and antibiotics, are all potential causes of AKI after liposuction [6]. It is critical to regularly monitor patients for signs of AKI following liposuction, particularly if they have a history of medical conditions such as hypertension or diabetes, which may increase their chance of developing AKI [6,7].

AKI might hinder liposuction recovery and require hospitalization and ICU monitoring. AKI patients may need dialysis or other therapies to maintain renal function and avert future complications. Aside from AKI, other potential side effects of liposuction include infection, bleeding, and skin problems [8-10].

The ADQI agreement on perioperative AKI provides critical advice on the prevention, diagnosis, and treatment of acute kidney damage after surgery [6]. These tips help healthcare professionals enhance patient care and improve surgical results. Perioperative AKI may be caused by a variety of factors, and determining the important factors in this specific patient's case would require thorough information on the patient and the surgical therapy [6,8]. However, given our extensive understanding of perioperative AKI, let us examine the likely origins and course of AKI, as well as strategies to prevent or reduce the risk of perioperative AKI [4,6,8].

Preoperative, intraoperative, and postoperative factors may all contribute to perioperative AKI [8]. Underlying chronic renal illness, diabetes mellitus, hypertension, advanced age, and certain medicines might all be risk factors for surgery. Intraoperative factors may include prolonged surgical duration, hypotension, hypovolemia, nephrotoxic medications, and significant blood loss [6,8]. Hypotension, hypovolemia, sepsis, and the need for critical care are all postoperative factors [8].

Perioperative AKI often begins with an insult during the perioperative phase, followed by a period of acute kidney injury and subsequent recovery [9]. The severity of AKI varies, ranging from mild kidney insufficiency to severe instances requiring renal replacement therapy [8,9]. Methods of prevention and risk reduction are required to reduce the occurrence of perioperative AKI. Various measures are supported by the ADQI consensus. Preoperative optimization involves diagnosing and treating preexisting conditions such as chronic kidney disease, diabetes, and hypertension before surgery to help enhance renal function [9].

Maintaining proper blood pressure and fluid status during surgery is crucial for achieving adequate renal perfusion [9,10]. Another critical component is medication management, which necessitates the adjustment or discontinuation of nephrotoxic medicines prior to surgery if suitable and the use of alternative therapies where available. Implementing enhanced recovery after surgery (ERAS) protocols may also be beneficial [10,11]. To reduce the risk of AKI, these techniques emphasize early mobility, adequate pain management, and fluid optimization. Furthermore, if possible, minimizing the use of nephrotoxic substances such as contrast media is recommended [10]. Close monitoring of urine production, serum creatinine levels, and other relevant indicators is essential for detecting early signs of AKI and acting quickly [3,4].

Perioperative AKI is a severe concern in surgical patients, and the ADQI consensus provides valuable preventative and treatment recommendations [6,8]. Identifying and managing potential risk factors via preoperative optimization, thorough intraoperative therapy, and watchful postoperative monitoring may help reduce the incidence and severity of perioperative AKI. Implementing prophylactic interventions and evidence-based recommendations may improve patient outcomes and reduce the prevalence of AKI in surgical settings [8,9].

The patient's difficulties demanded a collaborative effort from multiple medical teams, including the ICU, nephrology, surgery, anesthesia, and plastic surgery departments. The patient was first treated with a clear liquid diet, intravenous (IV) fluids, and pain relief. However, due to the severity of the problems, surgical intervention was eventually required.

The longer ICU stay was necessary to closely monitor the patient's vital signs and electrolyte levels. Therefore, the patient was also monitored for her elevated BUN, creatinine, and electrolyte levels. BUN and creatinine are markers of renal function, and high levels indicate impaired kidney function. Electrolytes are essential components in the body that participate in a variety of physiological activities. Electrolyte imbalances may be fatal, so they must be carefully monitored.

The patient's concerns in this case highlight the need for careful patient selection, thorough preoperative evaluation, and good intraoperative monitoring. Patients contemplating cosmetic surgery should be thoroughly evaluated to determine their overall health and suitability for the procedure. Adequate fluid resuscitation and monitoring after surgery may help minimize problems like hypovolemia and AKI. Moreover, AKI is a rare but possibly deadly liposuction complication [11,12]. To prevent and manage this outcome, individuals must be closely monitored, particularly those with preexisting medical difficulties. In the event of AKI, prompt intervention and care may be required to maintain renal function and avoid future complications.

This case study emphasizes the potential hazards associated with cosmetic surgery, particularly liposuction. The patient in this case had AKI, anemia, and a complicated hematoma, which required a collaborative effort from several medical teams and resulted in a difficult recovery and an extended ICU stay. To prevent these issues, cautious patient selection, preoperative evaluation, and proper intraoperative monitoring are required. Surgeons and medical teams should prioritize patient safety and take the appropriate steps to reduce the risk of complications associated with cosmetic surgery.

## Conclusions

This case report demonstrates the potential risks associated with aesthetic surgery, particularly liposuction. The patient had severe renal impairment, anemia, and a complex hematoma that required collaborative effort from many medical teams and a lengthy ICU stay. Following surgery, adequate fluid resuscitation and monitoring may help minimize complications such as hypovolemia and AKI. Patients seeking cosmetic surgery should undergo a thorough preoperative assessment to determine their overall health and suitability for the procedure. In the context of preoperative evaluation for liposuction candidates, specific tests can provide valuable information to identify individuals who may be at a higher risk of perioperative complications, including AKI. These tests may include a comprehensive assessment of renal function, including serum creatinine levels, estimated glomerular filtration rate (eGFR), and urinalysis. Additionally, preoperative evaluation should encompass a complete blood count (CBC) to assess hemoglobin levels and rule out anemia. Surgeons and medical teams should prioritize patient safety and follow adequate protocols to reduce the risk of complications associated with cosmetic surgery. In the case of AKI, prompt therapy and care may be required to maintain renal function and avoid complications. Conclusively, this case report emphasizes the need for careful patient selection, preoperative evaluation, and cautious intraoperative monitoring to ensure patient safety during cosmetic surgery.
